# G6f-Like Is an ITAM-Containing Collagen Receptor in Thrombocytes

**DOI:** 10.1371/journal.pone.0052622

**Published:** 2012-12-21

**Authors:** Craig E. Hughes, Uvaraj P. Radhakrishnan, Marie Lordkipanidzé, Stuart Egginton, Johannes M. Dijkstra, Pudur Jagadeeswaran, Stephen P. Watson

**Affiliations:** 1 Centre for Cardiovascular Sciences, Institute for Biomedical Research, The College of Medical and Dental Sciences, University of Birmingham, Edgbaston, Birmingham, United Kingdom; 2 Department of Biological Sciences, University of North Texas, Denton, Texas, United States of America; 3 Institute for Comprehensive Medical Science, Fujita Health University, Toyoake, Aichi, Japan; University of Edinburgh, United Kingdom

## Abstract

Collagen activates mammalian platelets through a complex of the immunoglobulin (Ig) receptor GPVI and the Fc receptor γ-chain, which has an immunoreceptor tyrosine-based activation motif (ITAM). Cross-linking of GPVI mediates activation through the sequential activation of Src and Syk family kinases and activation of PLCγ2. Nucleated thrombocytes in fish are activated by collagen but lack an ortholog of GPVI. In this study we show that collagen activates trout thrombocytes in whole blood and under flow conditions through a Src kinase driven pathway. We identify the Ig receptor G6f-like as a collagen receptor and demonstrate in a cell line assay that it signals through its cytoplasmic ITAM. Using a morpholino for *in vivo* knock-down of G6f-like levels in zebrafish, we observed a marked delay or absence of occlusion of the venous and arterial systems in response to laser injury. Thus, G6f-like is a physiologically relevant collagen receptor in fish thrombocytes which signals through the same ITAM-based signalling pathway as mammalian GPVI, providing a novel example of convergent evolution.

## Introduction

Mechanisms for the cessation of bleeding are an important evolutionary pressure, and complex organisms have all evolved haemostatic pathways. Studying human haemostatic and thrombotic disorders are possible due to the similarities between the pathways in mammals (i.e. mice); however less is known about the mechanisms in other species.

The mechanism of activation of mammalian platelets by the extracellular matrix protein collagen has been studied in great detail. In the high shear environment of the mammalian circulatory system, platelets are captured on von Willebrand factor (VWF)-coated collagen fibres through the GPIb-IX-V complex. Once arrested, the low affinity immunoglobulin (Ig) receptor, GPVI, binds to collagen and induces robust platelet activation though an immunoreceptor tyrosine-based activation motif (ITAM) in its associated Fc receptor γ-chain [1]. This is followed by the release of the powerful secondary agonists, ADP and thromboxane A_2_, allowing the recruitment of further platelets and the subsequent growth of a thrombus.

The use of zebrafish as a model organism for haemostasis and thrombosis has increased in recent years, even though we know much less about haemostasis in fish and there are significant differences to haemostasis in mammals. For example, fish thrombocytes are nucleated cells and are generally larger than the small, anucleate platelets of mammalian circulatory systems. Furthermore, orthologs of several key platelet receptors are absent in the fish genome, including the collagen receptor, GPVI, and the C-type lectin receptor, CLEC-2. Despite these differences, however, fish thrombocytes readily form thrombi in response to vessel wall injury and are activated by collagen, suggesting the presence of a novel collagen receptor [2], [3], [4], [5], [6], [7].

An ITAM has two Y*xx*Ls separated by 6–12 amino acids. Clustering of ITAM receptors such as the collagen receptor GPVI leads to Src family kinase-dependent phosphorylation of the two conserved tyrosines and subsequent binding and activation of Syk tyrosine kinase [1]. This initiates a signalling cascade that culminates in the activation of PLCγ2, which in turn mediates cellular activation through production of the secondary messengers inositol-1,4,5-trisphosphate (IP_3_) and 1,2-diacylglycerol (DAG). Syk is highly conserved throughout evolution and orthologs have been found in the first vertebrate, the jawless fish, which date back to the Cambrian era, over 500 million years ago [8].

We have been unable to identify a functional homologue of GPVI in non-mammalian vertebrates, including teleost fish. We therefore hypothesised that collagen activates fish thrombocytes in a similar pathway to that of mammals but through a novel ITAM receptor. A candidate for such a receptor is the protein known as G6f-like (G6fL) which has been shown to be expressed exclusively on thrombocytes, although interestingly it is also expressed on erythrocytes in crucian carp [9]. G6fL has four extracellular Ig-like domains, a single transmembrane domain and a short cytoplasmic tail containing a conserved Grb2 binding domain Y*x*N, and an ITAM [9]. The ITAM is split over two exons, a notable feature of classical ITAMs.

In the present study we demonstrate that G6fL is a functional receptor for collagen in thrombocytes in zebrafish, signalling through a similar pathway to the mammalian collagen receptor GPVI. This is a novel example of convergent evolution of the signalling pathway.

## Methods

### Reagents

Horm collagen was purchased from Nycomed (Munich, Germany). Human fibrinogen was purchased from Enzyme Research Laboratories (Swansea, UK). Horseradish-peroxidase-conjugated α-rabbit secondary antibody, and enhanced chemiluminescence reagents were purchased from Amersham Biosciences (GE Healthcare, Bucks, UK). DT40 cells [10] were provided by Dr M. Tomlinson (The University of Birmingham, UK). A polyclonal antibody for G6fL was produced by BioGenes (Berlin, Germany). Two rabbits were immunised with a peptide corresponding to residues 203–217 in the extracellular domain of zebrafish G6fL. Serum was tested by ELISA against the peptide and subsequently against zebrafish thrombocytes. All other reagents were purchased from Sigma-Aldrich (Poole, UK) or from previously described sources [11].

### Constructs

Zebrafish RNA was isolated with TRIzol from the anterior body part and then reverse transcribed into cDNA with random hexamers using the Superscript First-Strand Synthesis System for RT-PCR (Invitrogen, Paisley, UK). PCR was performed using PrimeSTAR HS DNA Polymerase (Takara Bio, Otsu, Japan) with the primers **G6FL-5′UTR-FWD** (5′-CTA-ACT-ATT-AAG-GAT-ATA-TTG-CTT-CCT-AGA-AGC-ATC-3′) and **G6FL-3′UTR-REV** (5′-TGT-CCC-ATT-ATT-TAT-GGA-CCT-AAC-TGT-AAC-3′). PCR fragments were blunt-end cloned into the HincII site of vector pUC118 and the sequence of individual clones were determined using CEQ Dye terminator Cycle Sequencing Kit (Beckman Coulter, High Wycombe, UK) and an automated sequencer CEQTM2000 DNA Analysis System (Beckman Coulter). The clone that was used for further experiment was a common splicoform identical with GenBank accession GU393012, except for an extra nucleotide triplet adding an alanine to the ectodomain stalk region [9].

G6fL was sub-cloned into the pEF6 expression vector using the following primers **G6fL-FWD** (5′-TAG-TAG-GGT-ACC-ACC-ATG-AGG-AAA-AAT-GAT-CAA-GCT-TTT-3′), **G6fL-REV** (5′-TAG-TAG-GCG-GCC-GCT-CAA-CCA-CAG-CAC-TGG-TTG-TAT-CTC-TCC-3′). Subsequent mutations were made using the Quikchange II Site Directed Mutagenesis kit (Agilent Technologies, Stockport, UK) with the following primers **G6fL-Y499F-FWD** (5′-CGG-AGA-GGT-GGA-GAA-CAT-CTT-TGA-AAA-TCC-TGA-TG-3′), **G6fL-Y499F-REV** (5′-CAT-CAG-GAT-TTT-CAA-AGA-TGT-TCT-CCA-CCT-CTC-CG-3′), **G6fL-Y515F-FWD** (5′-CCC-AAG-GCG-CAG-TCT-TCA-TGG-ATC-TCA-AGC-C-3′), **G6fL-Y515F-REV** (5′-GGC-TTG-AGA-TCC-ATG-AAG-ACT-GCG-CCT-TGG-G-3′), **G6fL-Y527F-FWD** (5′-CCA-ACG-GGT-GAA-ATG-GAT-GTA-TTT-AAA-GAA-TTG-GAG-AGA-TAC-AAC-3′), **G6fL-Y527F-REV** (5′-GTT-GTA-TCT-CTC-CAA-TTC-TTT-AAA-TAC-ATC-CAT-TTC-ACC-CGT-TGG-3′).

### Preparation of Thrombocytes from Trout Blood and Whole Blood Aggregometry

Blood was drawn from farmed rainbow trout (Hathersage, UK) in accordance with ‘The Humane Killing of Animals under Schedule 1 to the Animals (Scientific Procedures) Act 1986’ by concussion of the brain, into heparin (100 U/ml). Trout blood was kept on ice until required for study and all experiments were performed at room temperature. Anticoagulated blood from trout was diluted 1∶1 using normal saline (0.9%) in a mini-cuvette (Verum Diagnostica, Munich, Germany) and stirred for 3 min at room temperature. Dasatinib or DMSO vehicle control was pre-incubated at room temperature for 1 min followed by addition of collagen. Following agonist addition, aggregation was monitored at room temperature for 12 min, calculated by impedance across two electrode pairs (Multiplate analyser, Instrumentation Laboratory UK Ltd, Warrington, UK), and reported in arbitrary impedance units (AU).

### Static Adhesion Assay

Coverslips were coated with matrix proteins as previously reported [12] followed by blocking with 5 mg/ml of heat-inactivated bovine serum albumin (BSA) in PBS for 1 hr at room temperature. Trout thrombocytes were allowed to spread for 90 min at room temperature, before washing with PBS followed by fixation with paraformaldehyde (3.7%). Thrombocytes were imaged by DIC microscopy on a Zeiss Axiovert 200 M microscope.

### Flow Adhesion Assay

Trout blood was drawn into sodium heparin (100 U/ml). Glass capillary tubes (Camlab, Cambridge, UK) were coated with matrix proteins for 1 hr at room temperature followed by blocking with 5 mg/ml of heat-inactivated BSA in PBS for 1 hr at room temperature, and then mounted on the stage of an inverted microscope (DM IRB; Leica). Anticoagulated whole blood was pre-incubated with the fluorescent dye DiOC6 (3,3′-dihexyloxacarbocyanine iodide –2 µM) for 10 min before perfusion through the chamber at room temperature for 10 min. The thrombocytes were then washed with modified-Tyrodes buffer followed by imaging using DIC microscopy on a Zeiss Axiovert 200 M microscope.

### Western Blotting

Zebrafish larvae were lyophilised 48 hrs following injection and subsequently lysed in RIPA buffer with mechanical crushing to fully dissolve proteins. The equivalent of 4 larvae per well were separated by SDS-PAGE, electro-transferred, and western blotted. Transfected DT40 cells were lysed in RIPA buffer. 10^6^ cells per well were separated by SDS-PAGE, electro-transferred, and western blotted.

### Cell Culture and Transfection

DT40 chicken B-cells were grown in RPMI supplemented with 10% foetal bovine serum, 1% chicken serum, 100 U/ml penicillin, 100 µg/ml streptomycin, 50 µM β-mercaptoethanol and 20 mM GlutaMAX. Cells were transfected in 400 µl of serum-free media by electroporation using a GenePulser II (Bio-Rad, Hertfordshire, UK) set at 350 V and 500 µF. Cells were transfected as described above with either 10 µg of the indicated G6fL constructs, or with 2 µg of GPVI plus 2 µg of FcRγ, or with an appropriated empty vector control (Mock) and all cells with an additional 15 µg of the luciferase reporter construct [13], [14]. Twenty hours after transfection, live cells were counted by trypan blue exclusion. Luciferase assays were carried out as described previously [15]. Luciferase activity was measured with a Centro LB 960 microplate luminometer (Berthold Technologies, Germany).

### Zebrafish Knockdowns

Zebrafish (Ekkwell, Gibsonton, FL, USA) were maintained under standard conditions. An antisense G6fL Vivo-MO (5′-GCT-TGA-TCA-TTT-TTC-CTC-ATG-ATG-C-3′) against the zebrafish G6fL gene and a control Vivo-MO (5′-CCT-CTT-ACC-TCA-GTT-ACA-ATT-TAT-A-3′) were produced by Gene-Tools LLC (Philomath, OR, USA). Adult zebrafish (>3 months) were anesthetized and approximately 5 µl Vivo-MOs (either the original solution supplied by the vendor, 0.5 or 0.05 mM diluted with PBS, pH 7.4) were taken into a 27½ G needle such that the only Vivo-MO solution remained in the needle. For injection, the needle was placed into the region located between the second and third body stripes closer to the anal pore and at right angles to the location of the inferior vena cava. It was then gently inserted into the vessel and the contents immediately injected [16]. Zebrafish larvae (3 days post fertilisation (dpf)) were injected with 5–10 nl Vivo-MO with glass pulled needles and microinjected intravenously using a Nanoject II (Drummond, Broomall, PA, USA).

### Zebrafish Blood Sample Preparation and Aggregation

48 hrs post injection blood collection was performed by gently poking the lateral surface of the fish body where the caudal artery and the caudal vein anastomose with a needle. A micropipette set was used for collecting 1 µL blood welling out from the vessel. This 1 µl blood was immediately dispensed into a tube containing 1 µl of 3.8% sodium citrate in PBS. Thrombocyte aggregation was measured by adding 1 µl of citrated blood to 8 µl of 0.63% sodium citrate in PBS and either 1 µl ADP (200 µM) or collagen (1 mg/ml) in a conical well of a micro-titre plate. The plate was tilted manually every 5 min for 1–1.5 hrs at 25°C to determine the time taken to stop the flow of blood down the walls of the well, i.e. time taken for thrombocyte aggregation [7].

### Laser-induced Injury and Time to Occlusion Analysis

48 hrs following injection zebrafish larvae that showed normal blood circulation (late phenotype) were placed in agarose blocks and a pulsed nitrogen laser beam pumped through coumarin 440 dye (Micro Point Laser system, Photonic Instrument, St Charles, IL, USA) was used to injure vessels around 5–7 somites from the anal pore to measure time to occlusion as reported previously [17]. Time to occlusion was recorded up to 2 min after injury.

### Statistical Analysis

Where applicable, data is expressed as mean ± standard error. Statistical analysis was carried out using unpaired Student’s t-tests. Significance was taken P<0.05.

## Results

### Collagen Activates Trout Thrombocytes through a Src Kinase-dependent Pathway

We used blood from rainbow trout to provide a sufficient sample of blood for investigation of thrombocyte activation by collagen using whole blood impedance aggregometry (Multiplate), a method that is routinely used in the investigation of human platelets. Trout were one of the species of fish used in the initial identification of G6fL on thrombocytes and have previously been shown to be responsive to collagen, whereas leukocytes and erythrocytes are unresponsive to collagen [2], [9]. We were unable to investigate activation of thrombocytes using light transmission aggregometry as there is no straightforward way to separate thrombocytes from other blood cells due to their similar size.

**Figure 1:**
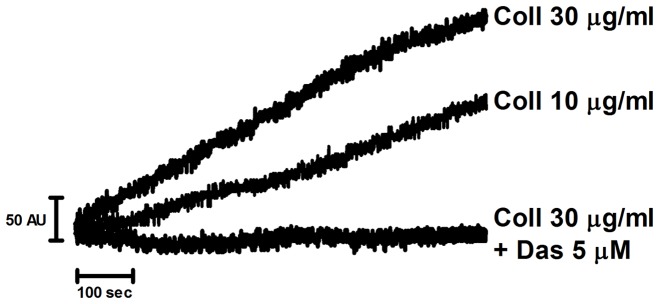
Trout thrombocytes aggregate to collagen in a Src dependent manner. Anticoagulated whole blood from rainbow trout was diluted 1∶1 with normal saline (0.9%) and stirred for 3 min at room temperature in a Multiplate mini-cuvette. Dasatinib (5 µM) or DMSO vehicle control was pre-incubated at room temperature for 1 min followed by addition of collagen, 30 or 10 µg/ml, and aggregation was monitored for 12 min at room temperature. Aggregation was measured in arbitrary impedance units (AU).

Collagen stimulates aggregation of trout thrombocytes at room temperature (Figure 1), which is approaching the upper temperature for trout. The response was similar to that in human platelets [18], although the time course was noticeably slower. The response to 30 µg/ml collagen was faster than that to 10 µg/ml indicating a similar concentration-response relationship to that seen in human platelets. Aggregation was inhibited by the Src kinase inhibitor dasatinib (5 µM) which is effective in whole blood in contrast to many other Src family kinase inhibitors [19]. This demonstrates that collagen stimulates aggregation rather than agglutination.

### Trout Thrombocytes Adhere to Collagen

**Figure 2:**
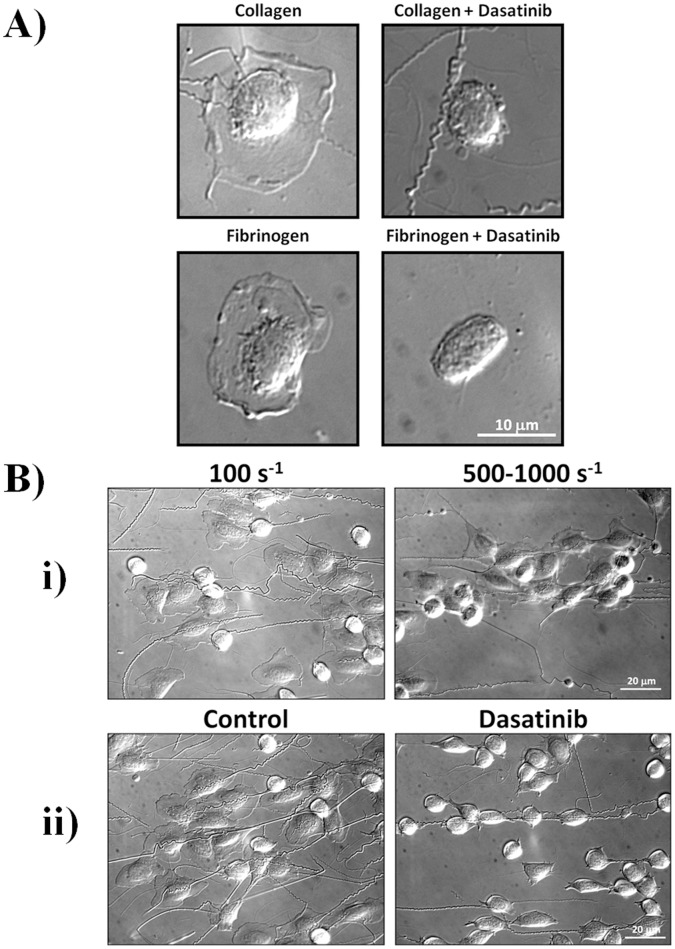
Trout thrombocytes adhere to and spread on collagen in a Src dependent manner. A) Trout blood was diluted by a factor of 100 to reduce the number of cells, and then allowed to adhere to either collagen or fibrinogen coated coverslips for 90 min at room temperature, either in the presence of 20 µM dasatinib or DMSO vehicle control. B) Anticoagulated whole blood was flowed through collagen coated glass capillary tubes at a range of shear rates (i) or at 100 s^−1^ for inhibitor studies (ii). Blood was pre-treated with either 20 µM dasatinib or DMSO vehicle control.

In trout blood, only thrombocytes bind to collagen [2] and thus they can be readily identified from other blood cells. Under static conditions, trout thrombocytes underwent adhesion and lamellipodia formation on collagen and also on fibrinogen-coated surfaces (Figure 2A). In both cases, spreading, but not adhesion, was completely inhibited by dasatinib (5 µM) consistent with a Src kinase mediated activation event. Furthermore, single thrombocytes were able to adhere and spread on collagen at shear rates ranging from 100–1000 s^−1^ (Figure 2B). In some instances, clusters of cells could be seen, although the number of cells in each cluster was small and they were present as a monolayer in contrast to the three dimensional aggregates observed in mammalian platelets under flow. The majority of the adherent cells in the clusters had formed lamellipodia which were inhibited in the presence of dasatinib. These results show that thrombocytes are able to adhere and spread on collagen under static and flow conditions, and that activation is blocked by Src family kinase inhibition. This result is similar to that seen in mammalian platelets where adhesion to collagen is not dependent on intracellular signalling as it is mediated by GPIb and α2β1 [20], but signalling through GPVI is required for spreading and aggregation.

### G6fL Responds to Collagen through an ITAM in a Src Dependent Manner

**Figure 3:**
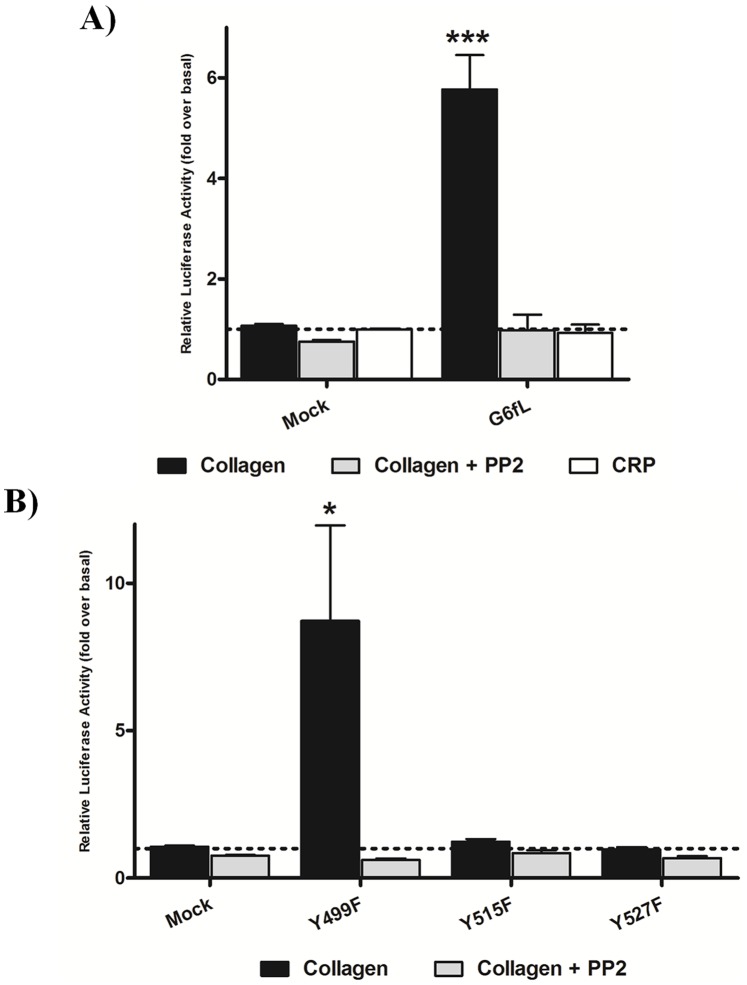
G6fL responds to collagen in a cell line assay through its ITAM. DT40 cells were transfected with wild type zebrafish G6fL (A) or with point mutants of G6fL (Y499F, Y515F and Y527F) (B) and stimulated with either collagen (10 µg/ml) or CRP (3 µg/ml). Where stated, cells were pre-incubated with 20 µM PP2. 6 hrs following stimulation, cells were lysed and luciferase activity was measured as a readout of signalling. Data is expressed as fold over basal (dotted line). Statistical significance was calculated with a Student’s t-test (*P>0.05, ***P>0.005). Similar levels of expression of all G6fL constructs was confirmed by western blotting (not shown).

The observation that collagen activates thrombocytes through a Src dependent pathway led us to search for a functional equivalent to the mammalian collagen receptor GPVI which is expressed in thrombocytes. The key signalling motif in the GPVI/FcR γ-chain complex is an ITAM, which has a conserved sequence of Y*xx*(L/I)x_6–12_Y*xx*(L/I), and initiates signalling through Src and Syk family kinases. A novel ITAM-containing receptor, G6fL, has recently been described on thrombocytes in teleost fish, and additionally on the erythrocytes of crucian carp, although its function is not known [9]. To investigate whether G6fL is a receptor for collagen, we used an NFAT-luciferase reporter assay that has been previously used to monitor activation of GPVI by collagen [21]. Zebrafish G6fL was cloned into an expression vector, and subsequently co-transfected into chicken DT40 B-cells along with an NFAT-luciferase reporter construct. Cells transfected with GPVI and FcR γ-chain expression vectors were used as a positive control. Six hours post stimulation luciferase activity was measured as a readout of receptor activation. Collagen (10 µg/ml) stimulated a robust signal in the GPVI/FcR γ-chain transfected cells, which was blocked by the Src kinase inhibitor PP2 (20 µM) (Figure S1), as previously reported [13]. Strikingly, collagen also stimulated a marked increase in luciferase activity in G6fL transfected cells compared to mock transfected cells (Figure 3A) and this response was again blocked by PP2, as expected for an ITAM signalling mechanism.

We used site-directed mutagenesis to determine the signalling motif that underlies activation. G6fL has two distinct signalling motifs, namely a Grb2 binding site, Y*x*N (Y499) and an ITAM with tyrosines at Y515 and Y527. Mutation of either of the conserved ITAM tyrosines to phenylalanine abrogated signalling to collagen (Figure 3B) thereby demonstrating that both tyrosines are required for signalling, consistent with an ITAM signalling pathway [11]. In contrast, mutation of the conserved tyrosine in the Grb2 binding site (Y499F) had no significant effect on the NFAT-reporter assay demonstrating that this tyrosine is not required for activation. These data demonstrate that G6fL requires an ITAM for signalling in response to collagen.

Activation of GPVI by collagen is mediated through a repeat glycine-proline-hydroxyproline (GPO) motif and synthetic triple helical peptides which contain this motif, known as collagen related peptides (CRP), are powerful activators of the Ig receptor (Figure S1) [22]. However, CRP (3 µg/ml) had no effect on G6fL-transfected cells demonstrating that collagen induces activation through a distinct collagen recognition sequence (Figure 3A). Consistent with this, CRP was unable to cause aggregation of trout thrombocytes (not shown) providing further evidence that G6fL recognising a distinct collagen motif. Thus, the motif in collagen for activation of G6fL is distinct to that for GPVI. The snake venom toxin convulxin, which also induces powerful activation of GPVI, also had no effect on G6fL-transfected cells (not shown) as anticipated given that they are encoded by distinct genes.

### G6fL is Required for Haemostasis in Zebrafish

**Figure 4:**
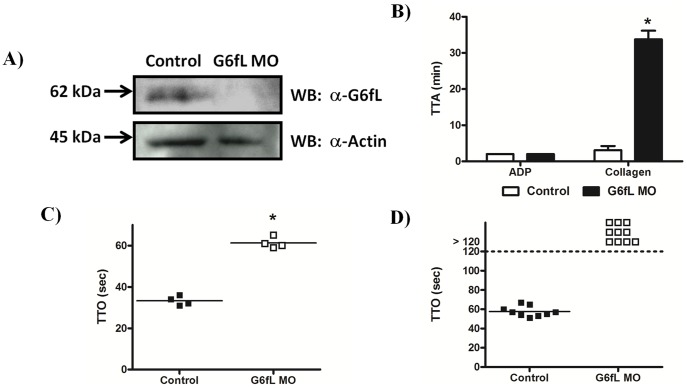
Zebrafish G6fL is required for haemostasis. A) A novel α-zebrafish G6fL antibody was used to determine knock-down of G6fL in morpholino (MO) treated larvae. 48 hrs post injection, 3 dpf larvae were lyophilised and lysed. Proteins were separated by SDS-PAGE and Western blotted with the antibody. The membrane was subsequently stripped and reprobed with an α-actin antibody to confirm equal loading. B) Control and G6fL MO treated adult fish were bled, and the blood used for a plate tilt aggregation assay. Time to aggregation (TTA) was measured following addition of either ADP (20 µM) or collagen (100 µg/ml). C) Control and G6fL MO treated larvae were immobilised on a microscope stage and then injured with a laser in the vessel wall of either veins or arteries (D). Time to occlusion (TTO) of vessels was measured for up to 2 min at which time the experiment was ceased. Statistical significance was calculated with a Student’s t-test (*P>0.05).

We used a Vivo-morpholino (MO) to knock-down G6fL levels *in vivo* in zebrafish to investigate a possible role for the receptor in the activation of thrombocytes and in haemostasis. A specific antibody to G6fL was raised and shown to detect a band at the predicted molecular weight of 59 kDa and this band was absent in the MO-treated larvae revealing a high degree of knock-down (Figure 4A). Equal loading was confirmed by reprobing the samples for actin. The ability of collagen to stimulate aggregation of thrombocytes in whole blood from zebrafish was measured using a plate-tilt assay, which measures the time taken for a drop of blood to aggregate following the addition of an agonist. The time to aggregation (TTA) by collagen in this assay is increased from less than 2 min in control MO-treated larvae to over 30 min in the G6fL MO-treated larvae, consistent with a critical role of G6fL in mediating thrombocyte activation (Figure 4B). In comparison, the time to aggregation in response to ADP was not altered. It was noted that the blood cell counts were not affected by the knock-down (not shown). These results confirm that G6fL is a collagen receptor on thrombocytes and that it plays a critical role in mediating thrombocyte activation by the matrix protein.

We used a laser injury assay to investigate the role of G6fL in thrombus formation *in vivo* in both arteries and veins using MO-treated larvae. We found that the G6fL MO-treated larvae took longer to occlude their veins compared to controls (Figure 4C) and that they were unable to occlude their arteries within the 120 sec experimental window (Figure 4D). This result confirms a critical role for G6fL in haemostasis in zebrafish.

## Discussion

In this study we have sought to understand the molecular mechanism of activation of fish thrombocytes by collagen. Zebrafish, a member of the teleost family, is an increasingly used model organism for mammalian haemostasis due to the speed and inexpensive nature of their use and genetic manipulation via morpholino technology. There are broad similarities which make this comparison possible to mammalian haemostasis such as the observation that thrombocytes will occlude vessels in response to an injury and that they have many orthologs of mammalian receptors. However, there are gaps in our understanding of fish thrombocytes, including the lack of a GPVI ortholog, although they do express an ortholog of the second mammalian collagen receptor, the integrin α2β1 complex [9]. The question therefore is whether this is sufficient to mediate platelet adhesion and activation by collagen or whether fish thrombocytes express a second receptor which mediates powerful activation, as is the case in mammalian platelets.

Here, we have shown that trout thrombocytes will respond to collagen in analogous ways to mammalian platelets i.e. via spreading and aggregation, and that this response is mediated by a Src family kinase. This is a new finding as the majority of the current literature on fish thrombocytes focuses on zebrafish, from which only tiny amount of blood can be removed for analysis. The collagen-binding integrin α2β1 has been reported at the gene level in teleost fish, and the integrin is known to signal weakly through Src in mammalian platelets although on its own this is insufficient to mediate platelet activation [20]. This raises the question as to whether activation of thrombocytes in fish is mediated solely through integrin α2β1 or whether they express a second activation receptor for collagen bearing in mind the absence of an ortholog of GPVI.

In the present study, we show that the novel ITAM-containing receptor G6fL, which has previously been shown to be expressed at the mRNA level in a number of teleost fish [9], functions as a collagen receptor in a cell line reporter assay and in zebrafish thrombocytes. G6fL has a number of similarities to the mammalian collagen receptor, the GPVI/FcR γ-chain complex, including the presence of extracellular Ig domains (four in the case of G6fL compared with 2 for GPVI) and a cytosolic ITAM which is similar to that on FcR γ-chain. In addition, it has sequence similarities to the Ig membrane protein G6f which is expressed on mammalian platelets, including the presence of a cytosolic Grb2-binding sequence, Y*x*N [9], [23].

We have shown that G6fL signals in response to collagen through a Src kinase dependent pathway that is critically dependent on the conserved tyrosines in its ITAM sequence but is independent of the tyrosine in the Grb2-binding sequence. The collagen sequence underlying activation of G6fL is distinct from that of GPVI in that it is unresponsive to CRP, which is a powerful ligand of the mammalian receptor. This suggests that although GPVI and G6fL are functionally equivalent in regard to activation of platelets/thrombocytes, they should not be considered as homologs, consistent with their structural differences and genomic locations [9]. This is an example of convergent evolution of the use of an ITAM to rapidly activate thrombocytes/platelets upon binding to collagen.

Definitive evidence that G6fL is a physiologically relevant collagen receptor mediating thrombocyte activation was achieved using an *in vivo* morpholino knock down approach in zebrafish. The *ex vivo* activation of platelets by collagen was markedly reduced following the knock-down of G6fL, leaving the response to ADP unaffected, and larvae were unable to occlude their arteries in a laser induced injury, while the response in the venous system was significantly delayed. While there is no direct evidence that the laser injury model exposes collagen, these results give strong indirect evidence that collagen is exposed as the result is reminiscent of that with GPVI or FcR γ-chain-deficient mice, which have abrogated platelet activation and marked reduction in thrombus formation, the extent of which is dependent on the *in vivo* assay injury [24], [25], [26]. However, we cannot rule out the possibility that although collagen is demonstrated here to be an agonist for G6fL, there may be other agonists *in vivo* which mediate thrombus formation.

G6fL is named after its mammalian ortholog G6f, although there are a significant number of differences between the two proteins including the reduced number of Ig domains and the absence of an ITAM in the mammalian protein. Furthermore, G6f does not appear to be a functional collagen receptor despite undergoing tyrosine phosphorylation following collagen or CRP treatment, as it is unable to support adhesion of transfected cells to a collagen surface or allow signalling to collagen of transfected cells [23]. The most striking similarities between the two proteins is the presence of the cytoplasmic Grb2-binding motif which suggests a novel pathway of regulation of Grb2-effector proteins including p42/44 MAP kinases, which in mammals play a critical role in platelet formation in and are implicated in the regulation of several platelet responses, including phosphorylation of cytosolic phospholipase A_2_ under certain conditions [27]. We hypothesise that if the mammalian receptor plays a signalling role in platelets it is either downstream of either a novel agonist or downstream of the collagen receptor GPVI.

In summary, in the present study, we have demonstrated the presence of a novel signalling receptor for collagen in teleost fish, G6fL, which plays an analogous role to mammalian GPVI. The structural features and gene location of G6fL and GPVI suggests that they are unlikely to be orthologs and it is therefore possible that these disparate receptors independently evolved to respond to collagen and signal through an ITAM allowing the rapid signalling required for thrombocyte/platelet activation and rapid haemostasis, an example of convergent evolution. Furthermore, as the mammalian ortholog of G6fL appears to not be a collagen receptor, this demonstrates divergent evolution.

## Supporting Information

Figure S1 **GPVI responds to collagen and CRP in a cell line assay.** DT40 cells were transfected with GPVI/FcRγ and stimulated with either collagen (10 µg/ml) or CRP (3 µg/ml). Where stated, cells were pre-incubated with 20 µM PP2. 6 hrs following stimulation, cells were lysed and luciferase activity was measured as a readout of signalling. Data is expressed as fold over basal (dotted line). Statistical significance was calculated with a Student’s t-test (*P>0.05, **P>0.01).
(TIF)
