# Supplementary figures and images for: G6f-Like Is an ITAM-Containing Collagen Receptor in Thrombocytes
Source: PLoS One. 2012 Dec 21;7(12):e52622. doi: 10.1371/journal.pone.0052622 (PMC3528668; doi:10.1371/journal.pone.0052622)

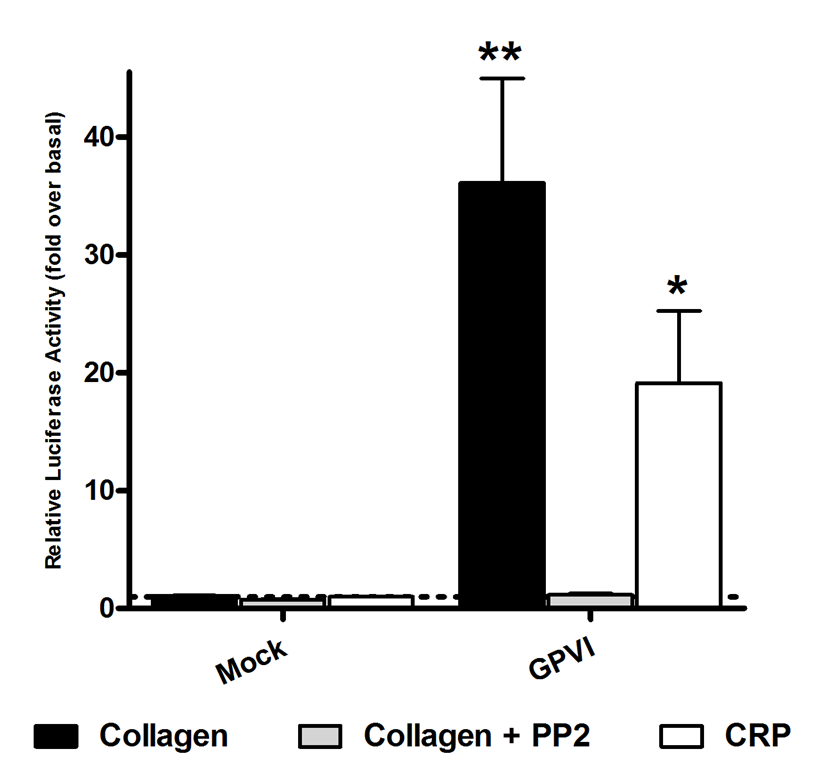

Supplement: Figure S1 — GPVI responds to collagen and CRP in a cell line assay. DT40 cells were transfected with GPVI/FcRγ and stimulated with either collagen (10 µg/ml) or CRP (3 µg/ml). Where stated, cells were pre-incubated with 20 µM PP2. 6 hrs following stimulation, cells were lysed and luciferase activity was measured as a readout of signalling. Data is expressed as fold over basal (dotted line). Statistical significance was calculated with a Student’s t-test (*P>0.05, **P>0.01). (TIF) [file pone.0052622.s001.tif]
